# Determination and prediction of standardized ileal amino acid digestibility of wheat bran in broiler chickens

**DOI:** 10.5713/ab.24.0003

**Published:** 2024-04-25

**Authors:** Xingbo Liu, Xianglong Yun, Zichen Cheng, Yuming Guo, Jianmin Yuan, Wei Nie

**Affiliations:** 1 National Key Laboratory of Animal Nutrition, College of Animal Science and Technology, China Agricultural University, 100193, Beijing, China

**Keywords:** Broiler, Prediction Equation, Standardized Ileal Amino Acid Digestibility, Wheat Bran

## Abstract

**Objective:**

The objective of this experiment was to determine the standard ileal digestibility (SID) of amino acid (AA) in 10 different sources of wheat bran fed to broilers and establish the SID AA prediction based on the chemical composition.

**Methods:**

A total of 660 1-day-old broilers were randomly divided into 11 treatments with 6 replicates of 10 chickens each. Diets included 10 semi-purified mash diets and 1 nitrogen-free diet. Titanium dioxide (TiO2) 0.50% was used as an indigestible index. On day 13, 6 chickens from each replicate were selected for slaughter to collect ileal contents. On day 28, 4 chickens from each replicate were selected for slaughter to collect ileal contents.

**Results:**

Results showed that the coefficient of variation of the conventional nutrients (except for gross energy, and dry matter) and all AAs was greater than 8.00%. The average SID of essential AA in wheat bran for 13-day-old broilers was 37.24% and the average SID of nonessential AA was 42.02%; the average SID of essential AA for 28-day-old broilers was 67.13% and the average SID of nonessential AA was 69.51%. A correlation was observed (p<0.05) between most SID AA and crude protein (CP), crude fiber (CF), acid detergent fiber (ADF), and ash at day 13. A correlation was observed (p<0.05) between most SID AA and CF, and ADF at day 28. The R^2^ value of stepwise regression equations for predicting the SID AA at day 13 and day 28 was best for glutamic acid (R^2^ = 0.97 using CP, ash, CF, ether extract (EE), and neutral detergent fiber [NDF]) and lysine (R^2^ = 0.74 using ash, ADF, EE, and NDF), respectively.

**Conclusion:**

In conclusion, broiler age had a significant effect on the SID AA values of wheat bran. The chemical composition of wheat bran varied widely between sources, and CP, CF, ADF, NDF, and ash were reasonable predictors of the SID AA of wheat bran.

## INTRODUCTION

Wheat bran is a by-product of wheat processing, comprising approximately 14% to 16% of the wheat [1]. It typically contains 55% to 60% non-starch carbohydrates, 14% to 25% starch, and 13% to 18% protein, among other constituents. Recognized as a high-value product, it finds extensive applications in the food, biofuel, livestock, health, and enzyme industries [1,2]. Studies have demonstrated that including wheat bran in poultry diets can enhance gizzard development and antioxidant capacity, improve nutrient digestibility [3], decrease ammonia emission from excreta [4], and mitigate salmonella colonization [5]. Moreover, given the high and fluctuating price of soybean meal, alternative feeds such as wheat bran may serve as valuable protein sources.

Accurate understanding of the energy and amino acid (AA) digestibility of wheat bran is essential for its effective utilization in dietary formulations. Standardized ileal digestibility (SID) of AA accounts for the basal endogenous losses (BEL) of AAs in the ileal digesta, derived from the apparent ileal digestibility (AID) of AAs, and is regarded as more dependable [6,7]. Formulating diets based on the SID AA values of ingredients allows for better alignment with the nutritional requirements of poultry and aids in reducing the nitrogen content of excreta [8]. Iyayi and Adeola [9] reported that in 26-day-old broiler chickens, the average SID of essential AA in wheat bran was 55.13%, while that of nonessential AA was 60.16%. However, Ullah et al [10] reported that the average SID of essential AA and nonessential AA in wheat bran were 77.94% and 75.50%, respectively, in 21-day-old broilers, which was similar to the values reported by Gallardo et al [11] in 21-day-old broilers. Sauvant et al [12] has conducted research on wheat bran as well and indicated that the average true amino acid digestibility of wheat bran in broilers was 77.18%. In previous research, while there have been valuable investigations into the digestibility of amino acids in broilers, certain limitations have been identified. For instance, many studies have focused primarily on broilers aged 21 to 28 days, thus lacking comprehensive data on amino acid digestibility in younger broilers aged 1 to 14 days [9–11]. Additionally, the selection of only one wheat bran for assessing amino acid digestibility in some studies may have introduced potential biases and limitations to their findings [9–11].

Additionally, the assessment of the SID AA through animal experiments is both cost-effective and time-intensive. Effective tools for estimating the SID AA have been developed through prediction equations, which rely on the correlation between the chemical composition of feed ingredients and the SID AA [13–16]. Thus, in the present study, 10 wheat bran samples from China were gathered and subsequently assessed for their SID AA values in broilers aged 13 and 28 days. Prediction equations for the SID AA of wheat bran, based on chemical composition, were established.

## MATERIALS AND METHODS

### Animal ethics statement

All study procedures were approved by the China Agriculture University Animal Care and Use Committee (AW11012202-1-3) and conducted under the Guidelines for Experimental Animals established by the Ministry of Science and Technology (Beijing, China).

### Wheat bran

The 1–10 wheat bran samples were collected from Shangqiu, Henan; Liaocheng, Shandong; Hengshui, Hebei; Beijing; Nanchang, Jiangxi; Jinan, Shandong; Handan, Hebei; Hefei, Anhui; Xuzhou, Jiangsu; and Zhoukou, Henan. The nutritional composition of wheat bran samples was presented and expressed on a dry matter (DM) basis (Table 1).

### Animals and management

A total of 660 one-day-old male Arbor Acres broilers were obtained from a local commercial hatchery (Gongzhuling, Jilin, China). Broilers were randomly divided into 11 treatments with 6 replicates of 10 chickens each. During the non-trial period, the broilers were provided with a commercial diet supplied by Liaoning Hefeng Herding Co., Ltd., Liaoning, China. Subsequently, the broilers were fed a semi-purified mash diet consisting of 91.40% wheat bran from days 10 to 13 and 25 to 28. Ileal contents were collected on days 13 and 28 for analysis.

### Diets and experimental design

The amino acid content of wheat bran was determined by the direct method and the experimental diet design was referenced by Yun et al [15]. Diets included 10 semi-purified mash diets and 1 nitrogen-free diet. Titanium dioxide (TiO_2_) at a concentration of 0.50% was utilized as an indigestible index, while the nitrogen-free diet served to determine the BEL AA. The ingredients of the experimental diets is shown in Table 2, and the amino acid content is shown in Table 3.

### Experimental procedures

On day 13, 6 chickens from each replicate were selected for slaughter. On day 28, 4 chickens from each replicate were selected for slaughter. The ileal contents of the broilers were collected following the study of Yun et al [15]. Briefly, broilers were euthanized by intravenous injection of sodium pentobarbital through their wings, and the contents of the distal ileum were collected by flushing with distilled water. Ileal contents from each replicate were pooled and stored at −20°C for subsequent studies. Samples were lyophilized, crushed, sieved, and stored at 4°C for amino acid analysis.

### Chemical analysis

As described by Liu et al [17], wheat bran samples were analyzed for the DM (AOAC, 2006 method 934.01), crude protein (CP, AOAC, 2006 method 990.03), ether extract (EE, AOAC, 2006 method 920.39), ash (AOAC, 2006 method 942.08), and starch (ST, AOAC, 2006 method 996.11) [18]. Additionally, gross energy (GE) was determined using an adiabatic bomb calorimeter (C 2000; IKA, Guangzhou, China). Neutral detergent fiber (NDF) and acid detergent fiber (ADF) of wheat bran samples were determined using an Ankom220 Fiber Analyzer (Ankom Technology, NY, USA). The AA content of test ingredients, test diets, and ileal digesta was analyzed by the Ministry of Agriculture Feed Potency and Safety Supervision, Inspection, and Testing Center (Beijing, China). Additionally, the TiO2 content of test diets and ileal digesta was determined following the method described by Short et al [19].

### Calculation

All data were expressed on a DM basis. The BEL, SID, and AID of AA were calculated using the equation described by Ghazaghi et al [20]:


$$\begin{array}{l} \text{BEL AA }(\text{mg}/\text{kg DMI})=\text{amino acid in ileal digesta }(\text{mg}/\text{kg})\times {\text{TiO}}_{2}\;\text{in diet }(\text{mg}/\text{kg})/{\text{TiO}}_{2}\;\text{in digesta }(\text{mg}/\text{kg}),\\ \text{AID AA }(\%)=[1-({\text{TiO}}_{2}\;\text{in diet }/{\text{TiO}}_{2}\;\text{in ileal digesta})\times (\text{amino acid in ileal digesta}/\text{amino acid in diet})],\\ \text{SID AA }(\%)=\text{AID AA }(\%)+[\text{BEL AA }(\text{mg}/\text{kg DMI})/(\text{amino acid content of the raw material }(\text{mg}/\text{kg DM})]\times 100, \end{array}$$

where DMI = dry matter intake.

### Statistical analysis

All data were analyzed by SPSS 22.0 (SPSS. Inc., Chicago, IL, USA). One-way analysis of variance followed by post-hoc multiple comparisons with Duncan’s multiple comparisons test was employed when a significant interaction was observed. The linear regression equations for predicting the SID AA values of wheat bran samples from the chemical constituents were calculated with the forward stepwise regression method within SPSS software. The results were expressed as mean values with their corresponding pooled standard error of the mean. Differences between means and interactions with p< 0.05 were considered statistically significant.

## RESULTS

### Chemical composition of wheat bran samples

The chemical and AA composition of wheat bran samples is presented in Table 1. The content of GE, DM, CP, ST, EE, ash, CF, NDF, and ADF were from 18.60 to 19.84 MJ/kg, 86.79% to 88.92%, 12.96% to 17.78%, 9.50% to 16.75%, 1.53% to 3.12%, 5.28% to 7.39%, 10.94% to 20.48%, 37.69% to 49.66%, and 10.82% to 21.73%, respectively. The coefficient of variation (CV) for these compositions, except for GE and DM, exceeded 8.00%. Regarding essential AA values, the concentrations of arginine (Arg), lysine (Lys), histidine (His), isoleucine (Ile), leucine (Leu), methionine (Met), phenylalanine (Phe), threonine (Thr), tryptophan (Trp), and valine (Val) ranged from 0.90% to 1.48%, 0.33% to 0.52%, 0.45% to 0.66%, 0.89% to 1.28%, 0.49% to 0.82%, 0.23% to 0.39%, 0.41% to 0.60%, 0.39% to 0.60%, 0.17% to 0.34%, and 0.63% to 0.94%, respectively. For nonessential AA values, the content of alanine (Ala), aspartic acid (Asp), cysteine (Cys), glutamic acid (Glu), glycine (Gly), proline (Pro), serine (Ser), and tyrosine (Tyr) ranged from 0.60% to 0.98%, 0.96% to 1.56%, 0.28% to 0.43%, 3.18% to 4.21%, 0.69% to 1.13%, 0.88% to 1.33%, 0.57% to 0.83%, and 0.33% to 0.53%, respectively. The CV of these amino acids ranged from 9.00 to 15.00%, except for Lys, where the CV was 25.64%.

### Standardized ileal digestibility of amino acids

Table 4 and 5 show the SID AA of the 10 wheat bran samples of 13-day-old and 28-day-old broilers, respectively. At day 13, the SID of Lys, Met, Phe, Thr, Trp, Cys, Glu, Pro, Ser, and Tyr was different (p<0.05) among the 10 wheat bran sources tested. The SID values of Met, Lys, Thr, and Trp ranged from 27.70% to 55.52%, 11.93% to 35.16%, 11.68% to 35.62%, and 30.89% to 53.20%, with averages of 43.09%, 22.17%, 21.96%, and 45.59%, respectively. Among the 10 essential AA, the highest mean SID AA was found for His (51.31%) and the lowest for Thr (21.96%), with the mean SID of the essential AA was 37.24%. Among the 8 nonessential AA, the highest mean SID AA was found for Glu (62.71%) and the lowest for Asp (32.12%), while the mean SID of the nonessential AA was 42.02%. At day 28, the SID of AA was different (p<0.01) among the 10 wheat bran sources tested. The SID values of Met, Lys, Thr, and Trp ranged from 56.30% to 87.93%, 31.05% to 83.36%, 32.52% to 78.97%, and 48.14% to 89.80%, with averages of 73.14%, 63.08%, 55.32%, and 72.46%, respectively. Among the 10 essential AA, the highest mean SID AA was found for Arg (77.13%) and the lowest for Thr (55.32%), with the mean SID of the essential AA was 67.13%. Among the 8 nonessential AA, the highest mean SID AA was found for Glu (82.45%) and the lowest for Ser (66.02%), with the mean SID of the nonessential AA was 69.51%.

The effect of broiler age on SID of amino acids of 10 wheat bran is shown in Table 6. The SID of the AA at 28 days was significantly higher (p<0.01) than the SID of the AA at 13 days among the 10 wheat bran sources tested. The mean SID for essential AA at 13 and 28 days were 37.24% and 67.13%, respectively, and for nonessential AA was 42.02% and 69.51%, respectively.

### Correlation analysis and the regression equations

The correlation between chemical composition and the SID AA of wheat bran samples is shown in Table 7 and 8. At day 13, the SID AA was negatively correlated (p<0.05) with the CP, except for Lys. A positive correlation (p<0.05) was found between the CF and ADF and the SID AA, except for Lys and Trp. There was a positive correlation (p<0.05) between the ash and the SID of Ile, Leu, Phe, Thr, Trp, Val, Asp, Cys, Ser, and Tyr, however, the SID of these amino acids, except for Phe and Val, were negatively correlated (p<0.05) with the EE. A positive correlation was observed (p<0.05) between the NDF and the SID of Thr, Trp, Arg, Lys, Asp, and Cys. At day 28, a negative correlation (p<0.05) was found between the CF, ADF and the SID AA, except for Arg, His, Leu, Phe, Glu, Gly, and Tyr. There was a positive correlation (p<0.05) between the ash and the SID of Tyr. Additionally, a negative correlation was observed (p<0.05) between the ST and the SID of Lys, Met, and Ala.

The linear regression equations predicting the SID AA at 13 and 28 days based on wheat bran chemical composition are shown in Table 9 and 10, respectively. At day 13, the R^2^ value of linear regression equations for predicting the SID AA was the best for Glu (R^2^ = 0.97 using CP, EE, ash, CF, and NDF), then followed by Phe (R^2^ = 0.94 using CP, EE, ash, and NDF), and least significant for Thr (R^2^ = 0.53 using CP, and EE) with intermediate values for the SID of Arg, His, Ile, Leu, Lys, Met, Trp, Val, Ala, Cys, Gly, Pro, Ser, and Tyr (R^2^ = 0.56 to 0.94; p<0.01). The best-fit equation for the SID of Met was SID Met = 82.47+16.86CF–12.71ADF+1.75CP–14.88ash–11.28EE (R^2^ = 0.89; p<0.01). The best-fit equation for the SID of Lys was SID Lys = 11.00+1.81NDF–10.44ash (R^2^ = 0.69; p<0.01). At day 28, the R^2^ value of linear regression equations for predicting the SID AA was the best for Lys (R^2^ = 0.74 using EE, ash, ADF, and NDF), then followed by Asp (R^2^ = 0.71 using EE, ash, ADF, and NDF), and least significant for Ile (R^2^ = 0.27 using CP, and ADF) with intermediate values for the SID of Met, Thr, Ala, Cys, Pro, and Tyr (R^2^ = 0.33 to 0.60; p<0.01). The best-fit equation for the SID of Met was SID Met = 231.67–3.97ADF–6.33CP (R^2^ = 0.36; p<0.01). The best-fit equation for the SID of Lys was SID Lys = −208.63–5.84ADF+3.59NDF+27.02EE+20.99ash (R^2^ = 0.74; p<0.01).

## DISCUSSION

### Chemical composition of wheat bran samples

The CV values of all chemical components of the 10 wheat brans in this study, except GE and DM, were greater than 8.00%. This suggests that the samples selected for testing may be representative. Among the conventional nutrients, apart from DM, the highest mean value was observed for NDF (44.23%), while the lowest value was noted for EE (2.08%). Additionally, the highest values of Arg (1.34%) were found in the essential amino acid (EAA), and the highest values of Glu (3.65%) in the nonessential amino acid (NEAA) in this study. These variations in chemical composition were similar to those reported by Trindade et al [21]. They observed that wheat bran sourced from Brazil exhibited the highest NDF (40.72%) and the lowest EE (3.15%) among the conventional nutrients, excluding DM. Additionally, the highest Arg (1.18%) among the EAA and the highest Glu (2.61%) among the NEAA were also noted in their study. It was also similar to the findings of Adedokun and Adeola [22], who reported that wheat bran sourced from the USA exhibited the highest NDF (49.84%) and the lowest EE (5.56%) among the conventional nutrients, excluding DM. Additionally, they noted the highest concentration of Glu (1.79%) among the NEAA. It is noteworthy that Glu content was found to be highest not only in wheat bran but also in corn, wheat, rice broken, sunflower meal, guar meal, and cottonseed meal [7,10,20]. Protein composition was likely significant in determining this property. Glutenin and alcohol-soluble proteins represented the primary protein fractions in wheat bran, both of which were characterized by elevated levels of Glu [23,24]. Variations in the chemical composition of wheat bran were also associated with factors such as cultivar diversity [15,25] and environmental conditions [26] during growth. Overall, the chemical composition of the samples was similar to the previously reported chemical composition of wheat bran [10,11,21,22].

### Standardized ileal digestibility of amino acids

The average SID AA of wheat bran at 13 and 28 days was 39.36% and 68.19%, respectively. Furthermore, the SID AA values were significantly higher in 28-day-old broilers (52.69% for Thr to 81.45% for Glu) than in 13-day-old broilers (20.82% for Thr to 62.39% for Glu), independent of EAA or NEAA. This suggests that the efficiency of broiler chickens in utilizing the amino acids in wheat bran increased with age. This trend was similar to that observed by Adedokun et al [27] in 5-day-old and 21-day-old broilers for corn distillers dried grains with solubles, and corn. Partly, this might be attributed to changes in the digestive system with age. As the broilers aged, pancreatic enzyme secretion increased, resulting in more effective promotion of protein digestion and hydrolysis in wheat bran [28,29]. Also, the microbiota tended to more mature and stabilize, which also affected the digestion of amino acids, resulting in a partial age effect [30].

There was no data on the amino acid digestibility of wheat bran in broilers aged 1 to 14 days and only sporadic data on the amino acid digestibility of wheat bran in broilers aged 21 to 28 days. Ullah et al [10] reported an average SID AA value of 76.90% for 1 wheat bran in 21-day-old broilers. Gallardo et al [11] investigated a mean SID amino acid content of 79.10% for 1 wheat bran in 21-day-old broilers. Sauvant et al [12] reported a SID AA of 77.18% for wheat bran in broilers. Their reported results were higher than the SID AA of 68.19% in this trial with 10 wheat bran in 28-day-old broilers. However, Iyayi and Adeola [9] reported a SID AA of 59.20% for 1 wheat bran in 26-day-old broilers, which was lower than the results reported in the present study. Amino acid digestibility was affected by a number of factors. Endogenous factors such as age [27], gender, and genotype [31] affected digestibility. Exogenous factors such as sample source [25], processing [16], and feeding conditions [32] could also affect digestibility. The differences in amino acid digestibility among studies may result from the combined effects of multiple factors.

### Correlation analysis and the regression equations

Multiple linear regression equations were used to predict the SID AA values in feed ingredients due to the correlation between the SID AA and the chemical composition of the sample [13]. The R^2^ values of the multiple regression equations predicting the SID AA on day 13 based on wheat bran composition ranged from 0.53 (Thr) to 0.97 (Glu), and on day 28 ranged from 0.27 (Ile) to 0.74 (Lys). Results of the multiple linear regression equations indicated that NDF, ADF, CF, CP, EE, and ash could be used as suitable predictors of the SID AA data for wheat bran. Correlation of the SID AA values with these nutrients found in this study was also reported in wheat [15], corn distillers dried grains with solubles [33], rapeseed meals [16], and soybean meals [14].

Also, a significant relationship between fibrous carbohydrates and most SID AA in wheat bran was found in this study. The results for the CF, and ADF of wheat bran were positively correlated with most SID AA at day 13 and negatively correlated with most SID AA at day 28. However, Wang et al [33] observed that the CF and ADF content of corn distillers dried grains with solubles exhibited negative correlations with some SID AA at day 14, while displaying positive correlations with some SID AA at day 28. These disparate findings could stem from the inherent distinctions between wheat bran and corn distillers dried grains with solubles as distinct feed ingredients, with their respective compositions potentially leading to variations in nutrient and amino acid digestibility. In addition, interactions between bran proteins and nonstarch polysaccharides (NSP) affected amino acid digestibility [11]. NSP or fiber intake increased the viscosity of intestinal chyme and limited the digestion and absorption of wheat bran proteins [34]. Therefore, fiber carbohydrates were effective predictors of the SID AA values in wheat bran.

Significant relationships between protein and amino acid digestibility have been reported several times [13,15,33]. The digestibility of proteins influenced the release and absorption of amino acids, while the digestibility of amino acids reflected the extent to which proteins were degraded and utilized in the digestive system [7,28]. The CP was significantly negatively correlated with the SID AA at 13 days in our study. This result was consistent with previous results. Wang et al [33] reported that a negative correlation between the CP and the SID AA in corn distillers dried grains with solubles. However, Yun et al [15] reported that the CP of wheat was positively correlated with the SID AA. The reason for this difference might have been the different protein compositions in wheat, wheat bran, and corn distillers dried grains with solubles. In addition, in the amino acid model, the inputs of EE and ash improved the predictive power of the equations. Although the amino acid digestibility of wheat bran might have been affected by a variety of factors, our results showed that fiber carbohydrates, CP, EE, and ash were closely related to the SID AA and could have been used as predictors of the SID AA in wheat bran.

## CONCLUSION

In conclusion, our study determined the average SID of EAA and NEAA in wheat bran for broilers aged 13 and 28 days. The average SID of EAA was found to be 37.24% for 13-day-old broilers and 67.13% for 28-day-old broilers, while the average SID of NEAA was 42.02% and 69.51%, respectively. Our findings underscore the significant relationship between the SID AA and the chemical composition of wheat bran, including CP, EE, ash, CF, and ADF. These results provide valuable insights into the nutritional value of wheat bran for broiler diets and offer a basis for developing predictive equations for the SID AA based on wheat bran’s chemical composition.

## Figures and Tables

**Table 1:** Analyzed energy and nutrient concentrations of wheat bran samples (%, as-DM basis)

Items	Sample numbers										Mean	Min	Max	CV^1)^ (%)

	1	2	3	4	5	6	7	8	9	10				
GE (MJ/kg)	19.47	18.95	18.92	19.05	19.10	18.83	18.93	19.84	18.60	18.80	19.05	18.60	19.84	1.88
DM	88.90	88.88	88.92	88.87	88.66	88.49	88.33	86.79	88.67	87.23	88.37	86.79	88.92	0.85
CP	17.65	17.10	17.43	16.74	15.73	15.92	16.68	17.78	12.96	14.15	16.21	12.96	17.78	9.74
ST	10.27	15.82	9.59	14.92	9.50	9.61	12.02	10.17	13.34	16.75	12.20	9.50	16.75	23.06
EE	3.12	1.95	1.68	2.56	2.62	1.85	1.56	1.70	1.53	2.25	2.08	1.53	3.12	25.73
Ash	5.28	6.26	6.40	6.32	6.87	6.96	6.85	6.75	7.39	6.90	6.60	5.28	7.39	8.72
CF	10.94	11.76	13.29	12.09	13.01	14.91	13.30	11.76	19.57	20.48	14.11	10.94	20.48	23.47
NDF	37.69	45.72	46.16	41.71	43.24	48.63	46.83	38.56	49.66	44.07	44.23	37.69	49.66	9.04
ADF	10.82	12.63	14.01	12.17	13.16	15.50	13.81	11.58	19.83	21.73	14.52	10.82	21.73	24.63
Essential amino acids														
Arg	1.45	1.48	1.40	1.44	1.40	1.39	1.36	1.36	0.90	1.23	1.34	0.90	1.48	12.61
His	0.52	0.51	0.48	0.50	0.50	0.48	0.46	0.49	0.33	0.43	0.47	0.33	0.52	11.83
Ile	0.66	0.59	0.59	0.61	0.58	0.56	0.55	0.62	0.45	0.55	0.58	0.45	0.66	9.69
Leu	1.28	1.13	1.16	1.21	1.14	1.13	1.07	1.23	0.89	1.11	1.14	0.89	1.28	9.34
Lys	0.82	0.79	0.77	0.80	0.77	0.78	0.70	0.72	0.49	0.69	0.73	0.49	0.82	13.08
Met	0.35	0.39	0.27	0.33	0.30	0.35	0.32	0.34	0.31	0.23	0.32	0.23	0.39	14.19
Phe	0.60	0.55	0.54	0.57	0.54	0.52	0.50	0.57	0.41	0.52	0.53	0.41	0.60	9.71
Thr	0.60	0.57	0.54	0.57	0.54	0.54	0.51	0.59	0.39	0.52	0.53	0.39	0.60	11.08
Trp	0.21	0.31	0.34	0.29	0.34	0.34	0.33	0.24	0.17	0.17	0.27	0.17	0.34	25.64
Val	0.94	0.87	0.85	0.85	0.84	0.82	0.79	0.87	0.63	0.79	0.82	0.63	0.94	9.84
Nonessential amino acids														
Ala	0.98	0.93	0.85	0.88	0.86	0.84	0.80	0.86	0.60	0.78	0.84	0.60	0.98	12.11
Asp	1.56	1.53	1.47	1.49	1.51	1.50	1.43	1.42	0.96	1.26	1.41	0.96	1.56	12.70
Cys	0.38	0.42	0.37	0.43	0.39	0.41	0.39	0.42	0.35	0.28	0.39	0.28	0.43	11.49
Glu	4.18	3.69	3.58	3.81	3.53	3.50	3.18	4.21	3.20	3.61	3.65	3.18	4.21	9.53
Gly	1.13	1.09	1.02	1.05	1.05	1.04	1.00	1.03	0.69	0.94	1.00	0.69	1.13	12.08
Pro	1.33	1.23	1.23	1.25	1.18	1.17	1.14	1.21	0.88	1.07	1.17	0.88	1.33	10.51
Ser	0.83	0.79	0.76	0.80	0.76	0.77	0.72	0.83	0.57	0.71	0.75	0.57	0.83	10.09
Tyr	0.53	0.47	0.51	0.49	0.44	0.48	0.44	0.51	0.33	0.42	0.46	0.33	0.53	12.59

Min, minimum; Max, maximum; GE, gross energy; DM, dry matter; CP, crude protein; EE, ether extract; CF, crude fiber; NDF, neutral detergent fiber; ADF, acid detergent fiber; ST, starch; Arg, Arginine; His, Histidine; Ile, Isoleucine; Leu, Leucine; Lys, Lysine; Met, Methionine; Phe, Phenylalanine; Thr, Threonine; Trp, Tryptophan; Val, Valine; Ala, Alanine; Asp, Aspartic acid; Cys, Cystine; Glu, Glutamic acid; Gly, Glycine; Pro, Proline; Ser, Serine; Tyr, Tyrosine.

1) Coefficient of variation (CV, %) = (stand deviation/mean) × 100%.

**Table 2:** Ingredients composition of the experimental diets (%, as-fed basis)

Ingredient	Wheat bran	N-free diet
Test ingredient	91.40	0.00
Starch	0.00	59.88
Sucrose	0.00	31.50
Cellulose	0.00	4.20
Soybean oil	4.00	0.00
CaCO_3_	0.00	1.30
Dicalcium phosphate	1.90	1.90
Stone power	1.30	0.00
NaCl	0.30	0.30
Choline chloride (60%)	0.20	0.20
Vitamin premix^1)^	0.20	0.02
Mineral premix^2)^	0.20	0.20
Titanium dioxide	0.50	0.50
Total	100.00	100.00

1) Provided per kg of diet: vitamin A, 12,500 IU; vitamin D_3_, 3,500 IU; vitamin E, 20 IU; vitamin K_3_, 3 mg; vitamin B_1_, 0.01 mg; vitamin B_2_, 8.00 mg; vitamin B_6_, 4.5 mg; vitamin B_12_, 0.02 mg; nicotinic acid, 34 mg; pantothenic acid, 12 mg; folic acid, 0.5 mg; biotin, 0.2 mg.

2) Provided per kg of diet: Fe, 80 mg; Cu, 8 mg; Zn, 80 mg; Mn, 80 mg; I, 0.7 mg; Se, 0.3 mg.

**Table 3:** The concentration of amino acids in the semi-purified mash experimental diets (%, as-fed basis)

Items	Wheat bran diet numbers									

	1	2	3	4	5	6	7	8	9	10
Essential amino acids										
Arg	1.14	1.17	1.15	1.03	1.10	1.12	1.06	1.11	0.92	0.75
His	0.45	0.45	0.44	0.41	0.44	0.43	0.41	0.42	0.36	0.31
Ile	0.53	0.49	0.49	0.48	0.48	0.48	0.45	0.53	0.42	0.40
Leu	0.98	0.91	0.91	0.90	0.91	0.89	0.85	0.99	0.80	0.76
Lys	0.75	0.73	0.70	0.66	0.68	0.68	0.63	0.64	0.58	0.47
Met	0.27	0.23	0.25	0.25	0.22	0.23	0.22	0.26	0.22	0.18
Phe	0.66	0.61	0.62	0.60	0.63	0.60	0.58	0.69	0.54	0.53
Thr	0.54	0.51	0.51	0.50	0.49	0.49	0.48	0.52	0.44	0.38
Trp	0.23	0.29	0.28	0.27	0.27	0.28	0.28	0.22	0.19	0.15
Val	0.81	0.77	0.76	0.74	0.74	0.75	0.72	0.81	0.67	0.58
Nonessential amino acids										
Ala	0.81	0.79	0.77	0.76	0.77	0.76	0.74	0.79	0.68	0.52
Asp	1.20	1.22	1.19	1.14	1.17	1.16	1.14	1.14	0.97	0.82
Cys	0.32	0.32	0.32	0.31	0.29	0.31	0.30	0.31	0.27	0.24
Glu	3.38	2.94	2.97	2.92	2.77	2.84	2.64	3.54	2.68	2.65
Gly	0.89	0.89	0.87	0.84	0.86	0.86	0.85	0.88	0.74	0.61
Pro	1.06	0.89	0.90	0.92	0.84	0.87	0.79	1.12	0.85	0.84
Ser	0.70	0.67	0.66	0.64	0.64	0.63	0.61	0.70	0.57	0.51
Tyr	0.40	0.36	0.38	0.36	0.43	0.37	0.36	0.40	0.33	0.34

Arg, Arginine; His, Histidine; Ile, Isoleucine; Leu, Leucine; Lys, Lysine; Met, Methionine; Phe, Phenylalanine; Thr, Threonine; Trp, Tryptophan; Val, Valine; Ala, Alanine; Asp, Aspartic acid; Cys, Cystine; Glu, Glutamic acid; Gly, Glycine; Pro, Proline; Ser, Serine; Tyr, Tyrosine.

**Table 4:** Standardized ileal digestibility of amino acids of 10 wheat bran samples of 13-day-old broilers (%, as DM basis)

Items	Sample numbers										SEM	p-value	Average	CV^1)^ (%)

	1	2	3	4	5	6	7	8	9	10				
Essential amino acids														
Arg	50.10	49.47	46.54	46.59	50.56	46.52	47.74	43.37	55.50	52.57	1.07	0.30	48.86	15.97
His	57.48	50.63	50.40	51.04	51.82	47.82	51.36	43.32	57.77	54.65	1.09	0.05	51.31	15.52
Ile	27.70	28.30	25.30	21.41	28.57	27.21	29.67	35.70	42.35	36.26	1.68	0.21	31.11	39.26
Leu	32.74	32.70	29.75	27.00	32.36	29.86	32.26	39.00	44.82	40.38	1.53	0.20	34.78	32.00
Lys	29.22^ab^	30.68^ab^	22.23^abc^	16.96^bc^	19.14^bc^	22.39^abc^	22.83^abc^	11.93^c^	35.16^a^	10.43^c^	1.69	<0.01	22.17	55.57
Met	46.46^ab^	36.01^bc^	42.52^ab^	39.39^bc^	27.70^c^	45.48^ab^	38.19^bc^	45.36^ab^	55.52^a^	47.77^ab^	1.57	0.01	43.09	26.48
Phe	39.22^b^	34.53^b^	35.02^b^	39.54^b^	46.05^ab^	40.41^b^	43.32^ab^	45.13^ab^	54.39^a^	54.24^a^	1.42	<0.01	43.65	23.65
Thr	12.26^c^	15.75^bc^	15.23^bc^	26.91^ab^	11.68^c^	24.13^abc^	22.21^bc^	21.17^bc^	35.62^a^	23.27^bc^	1.46	<0.01	21.96	46.22
Trp	30.89^c^	42.33^abc^	43.84^abc^	53.20^a^	49.05^ab^	50.31^ab^	50.45^ab^	41.98^abc^	54.13^a^	38.52^bc^	1.45	<0.01	45.59	23.14
Val	32.78	29.14	27.81	26.82	32.77	31.10	35.46	35.59	44.24	39.42	1.39	0.13	34.15	29.65
Mean	35.89	34.95	33.86	34.89	34.97	36.52	37.35	36.25	47.95	39.75	1.19	0.30	37.24	32.04
Nonessential amino acids														
Ala	35.07	31.69	29.37	29.16	32.77	30.37	34.45	31.89	43.60	31.86	1.30	0.38	33.29	28.37
Asp	28.99	30.31	28.47	25.63	33.12	30.66	35.98	28.80	42.59	32.29	1.46	0.36	32.12	33.07
Cys	44.27^ab^	41.13^b^	49.32^ab^	40.55^b^	40.56^b^	46.72^ab^	49.17^ab^	46.06^ab^	54.91^a^	55.69^a^	1.21	0.02	47.31	18.65
Glu	65.15^ab^	60.19^b^	59.63^b^	59.06^b^	59.16^b^	57.81^b^	59.08^b^	65.86^ab^	68.68^a^	69.28^a^	0.93	<0.01	62.71	10.80
Gly	40.41	34.64	33.89	36.82	39.43	31.86	38.34	32.63	45.19	40.58	1.23	0.25	37.24	24.10
Pro	59.63^ab^	53.37^b^	51.97^b^	51.82^b^	52.82^b^	53.08^b^	53.25^b^	64.93^a^	63.43^a^	63.42^a^	1.09	<0.01	57.37	13.80
Ser	28.60^bc^	27.43^bc^	26.48^bc^	22.08^c^	29.30^bc^	28.60^bc^	34.02^abc^	36.28^abc^	46.06^a^	38.70^ab^	1.64	0.04	32.72	36.53
Tyr	23.33^c^	21.62^c^	26.43^c^	33.23^c^	50.79^ab^	32.40^c^	36.72^bc^	33.61^c^	50.16^ab^	54.92^a^	2.05	<0.01	36.35	40.98
Mean	40.68	37.55	38.20	37.29	42.24	38.94	42.63	42.51	51.83	48.34	1.43	0.36	42.02	30.49

SEM, standard error of the mean; Arg, Arginine; His, Histidine; Ile, Isoleucine; Leu, Leucine; Lys, Lysine; Met, Methionine; Phe, Phenylalanine; Thr, Threonine; Trp, Tryptophan; Val, Valine; Ala, Alanine; Asp, Aspartic acid; Cys, Cystine; Glu, Glutamic acid; Gly, Glycine; Pro, Proline; Ser, Serine; Tyr, Tyrosine.

1) Coefficient of variation (CV, %) = (stand deviation/mean) × 100%.

a–c Different superscripts in each row for each factor differ significantly (p<0.05).

**Table 5:** Standardized ileal digestibility of amino acids of 10 wheat bran samples of 28-day-old broilers (%, as DM basis)

Items	Sample numbers										SEM	p-value	Average	CV^1)^ (%)

	1	2	3	4	5	6	7	8	9	10				
Essential amino acids														
Arg	70.08^c^	63.37^de^	69.54^cd^	89.36^ab^	90.64^a^	83.93^b^	93.33^a^	60.22^e^	68.85^cd^	63.24^de^	1.71	<0.01	77.13	16.91
His	67.81^cd^	60.23^e^	69.50^c^	85.55^a^	85.46^a^	76.52^b^	89.51^a^	60.00^e^	67.27^cd^	62.18^de^	1.56	<0.01	73.99	16.03
Ile	63.52^cd^	56.95^d^	59.71^d^	79.96^ab^	84.65^a^	71.24^bc^	87.83^a^	55.51^d^	56.37^d^	53.16^d^	1.92	<0.01	68.90	21.24
Leu	62.76^cd^	54.88^d^	60.67^cd^	78.61^ab^	83.06^a^	69.76^bc^	86.55^a^	55.75^d^	58.06^d^	55.46^d^	1.79	<0.01	68.33	19.96
Lys	58.95^cd^	53.87^de^	56.03^cde^	79.02^ab^	82.03^a^	67.07^bc^	83.36^a^	44.57^e^	45.43^e^	31.05^f^	2.45	<0.01	63.08	29.60
Met	70.14^bc^	60.61^de^	66.66^bcd^	85.25^a^	85.94^a^	74.46^b^	87.93^a^	63.99^cde^	62.46^cde^	56.30^e^	1.65	<0.01	73.14	17.21
Phe	60.85^c^	51.21^d^	65.38^c^	84.09^ab^	87.80^a^	77.33^b^	90.36^a^	62.46^c^	64.57^c^	63.84^c^	1.89	<0.01	72.21	19.93
Thr	52.46^cde^	45.44^def^	46.09^def^	63.00^bc^	74.31^ab^	57.65^cd^	78.97^a^	34.12^f^	42.34^ef^	32.52^f^	2.33	<0.01	55.32	32.14
Trp	60.03^d^	63.78^d^	72.44^c^	86.18^ab^	87.29^ab^	81.81^b^	89.80^a^	51.43^e^	61.10^d^	48.14^e^	2.01	<0.01	72.46	21.09
Val	61.79^c^	53.75^cde^	57.62^cd^	79.33^ab^	84.18^a^	72.71^b^	87.55^a^	49.57^de^	55.92^cde^	47.66^e^	2.03	<0.01	67.33	22.94
Mean	62.84^c^	56.41^cd^	62.36^c^	81.03^a^	84.54^a^	73.25^b^	87.52^a^	53.76^d^	58.24^cd^	51.35^d^	1.47	<0.01	67.13	21.84
Nonessential amino acids														
Ala	60.31^c^	52.73^cd^	57.54^c^	81.51^ab^	85.74^a^	74.91^b^	87.70^a^	48.21^de^	55.74^cd^	41.66^e^	2.21	<0.01	67.22	25.01
Asp	60.73^c^	55.76^cd^	58.15^cd^	81.53^ab^	86.17^a^	74.88^b^	88.56^a^	49.15^de^	54.95^cd^	45.27^e^	2.16	<0.01	68.03	24.16
Cys	64.65^c^	61.17^cd^	67.47^bc^	82.76^a^	82.42^a^	73.01^b^	87.19^a^	62.76^cd^	62.56^cd^	56.92^d^	1.51	<0.01	71.61	16.06
Glu	80.14^bc^	73.47^d^	76.33^cd^	89.22^a^	90.68^a^	83.93^b^	92.26^a^	75.99^cd^	76.45^cd^	75.98^cd^	1.02	<0.01	82.45	9.43
Gly	58.22^cd^	50.45^d^	59.96^c^	82.96^ab^	87.68^a^	77.72^b^	88.05^a^	52.07^cd^	59.03^c^	50.59^d^	2.12	<0.01	68.84	23.45
Pro	77.61^bcd^	68.80^e^	70.75^de^	78.74^bc^	81.68^ab^	71.84^cde^	86.22^a^	71.98^cde^	71.78^cde^	68.13^e^	1.03	<0.01	75.63	10.38
Ser	61.49^cd^	54.15^de^	57.01^de^	76.49^ab^	82.19^a^	69.49^bc^	85.05^a^	49.73^e^	54.86^de^	48.08^e^	1.94	<0.01	66.02	22.37
Tyr	54.33^e^	42.33^f^	65.93^cd^	81.44^ab^	86.57^a^	73.76^bc^	87.86^a^	60.76^de^	68.45^cd^	69.95^cd^	2.09	<0.01	70.05	22.75
Mean	64.68^c^	57.36^c^	64.14^c^	81.83^ab^	85.39^a^	74.94^b^	87.86^a^	58.83^c^	62.98^c^	57.07^c^	1.53	<0.01	69.51	19.62

SEM, standard error of the mean; Arg, Arginine; His, Histidine; Ile, Isoleucine; Leu, Leucine; Lys, Lysine; Met, Methionine; Phe, Phenylalanine; Thr, Threonine; Trp, Tryptophan; Val, Valine; Ala, Alanine; Asp, Aspartic acid; Cys, Cystine; Glu, Glutamic acid; Gly, Glycine; Pro, Proline; Ser, Serine; Tyr, Tyrosine.

1) Coefficient of variation (CV, %) = (stand deviation/mean) × 100%.

a–f Different superscripts in each row for each factor differ significantly (p<0.05).

**Table 6:** Standardized ileal digestibility of amino acids of 10 wheat bran samples in broiler chickens at 13 and 28 days of age (%, as DM basis)

Items	13 d	28 d	SEM	p-value
Essential amino acids				
Arg	48.90^b^	75.26^a^	3.64	<0.01
His	51.63^b^	72.40^a^	3.01	<0.01
Ile	30.25^b^	66.89^a^	4.75	<0.01
Leu	34.09^b^	66.56^a^	4.25	<0.01
Lys	22.10^b^	60.14^a^	5.28	<0.01
Met	42.44^b^	71.37^a^	3.94	<0.01
Phe	43.19^b^	70.79^a^	3.91	<0.01
Thr	20.82^b^	52.69^a^	4.53	<0.01
Trp	45.47^b^	70.20^a^	3.87	<0.01
Val	33.51^b^	65.01^a^	4.35	<0.01
Mean	37.24^b^	67.13^a^	4.05	<0.01
Nonessential amino acids				
Ala	33.02^b^	64.61^a^	4.47	<0.01
Asp	31.68^b^	65.52^a^	4.64	<0.01
Cys	46.84^b^	70.09^a^	3.24	<0.01
Glu	62.39^b^	81.45^a^	2.53	<0.01
Gly	37.38^b^	66.67^a^	4.18	<0.01
Pro	56.77^b^	74.75^a^	2.41	<0.01
Ser	31.76^b^	63.85^a^	4.37	<0.01
Tyr	36.32^b^	69.14^a^	4.73	<0.01
Mean	42.02^b^	69.51^a^	3.72	<0.01

SEM, standard error of the mean; Arg, Arginine; His, Histidine; Ile, Isoleucine; Leu, Leucine; Lys, Lysine; Met, Methionine; Phe, Phenylalanine; Thr, Threonine; Trp, Tryptophan; Val, Valine; Ala, Alanine; Asp, Aspartic acid; Cys, Cystine; Glu, Glutamic acid; Gly, Glycine; Pro, Proline; Ser, Serine; Tyr, Tyrosine.

a,b Different superscripts in each row for each factor differ significantly (p<0.05).

**Table 7:** The correlation coefficient between chemical composition and standardized ileal digestibility of amino acid of wheat bran samples in 13-day-old broilers (%, as DM basis)

Items	CP	ST	EE	Ash	CF	NDF	ADF
Essential amino acids							
Arg	−0.81^**^	0.42^*^	0.13	0.23	0.68^**^	0.36^*^	0.67^**^
His	−0.53^**^	0.31	0.38^*^	−0.17	0.43^*^	0.14	0.41^*^
Ile	−0.67^**^	0.19	−0.42^*^	0.57^**^	0.69^**^	0.21	0.65^**^
Leu	−0.65^**^	0.26	−0.32^*^	0.48^**^	0.68^**^	0.12	0.64^**^
Lys	−0.15	−0.04	−0.09	−0.15	−0.04	0.41^*^	−0.05
Met	−0.43^*^	0.14	−0.28	0.16	0.57^**^	0.16	0.53^**^
Phe	−0.83^**^	0.26	−0.13	0.64^**^	0.81^**^	0.16	0.76^**^
Thr	−0.66^**^	0.39^*^	−0.49^**^	0.63^**^	0.63^**^	0.48^**^	0.59^**^
Trp	−0.40^*^	0.02	−0.48^**^	0.70^**^	0.21	0.63^**^	0.19
Val	−0.75^**^	0.18	−0.3	0.55^**^	0.74^**^	0.23	0.69^**^
Nonessential amino acids							
Ala	−0.62^**^	0.06	−0.2	0.31	0.46^*^	0.29	0.40^*^
Asp	−0.76^**^	0.09	−0.45^*^	0.64^**^	0.64^**^	0.61^**^	0.61^**^
Cys	−0.65^**^	0.21	−0.50^**^	0.50^**^	0.85^**^	0.44^*^	0.84^**^
Glu	−0.50^**^	0.34^*^	0.00	0.12	0.60^**^	−0.19	0.56^**^
Gly	−0.69^**^	0.32	0.23	0.14	0.54^**^	0.11	0.49^**^
Pro	−0.37^*^	0.17	−0.08	0.18	0.48^**^	−0.27	0.42^*^
Ser	−0.71^**^	0.17	−0.46^*^	0.61^**^	0.74^**^	0.29	0.69^**^
Tyr	−0.82^**^	0.22	−0.07	0.71^**^	0.77^**^	0.26	0.73^**^

CP, crude protein; ST, starch; EE, ether extract; CF, crude fiber; NDF, neutral detergent fiber; ADF, acid detergent fiber; Arg, Arginine; His, Histidine; Ile, Isoleucine; Leu, Leucine; Lys, Lysine; Met, Methionine; Phe, Phenylalanine; Thr, Threonine; Trp, Tryptophan; Val, Valine; Ala, Alanine; Asp, Aspartic acid; Cys, Cystine; Glu, Glutamic acid; Gly, Glycine; Pro, Proline; Ser, Serine; Tyr, Tyrosine.

* p<0.05,

** p<0.01.

**Table 8:** Correlation coefficient between chemical composition and standardized ileal digestibility of amino acid of wheat bran samples in 28-day-old broilers (%, as DM basis)

Items	CP	ST	EE	Ash	CF	NDF	ADF
Essential amino acids							
Arg	0.02	−0.24	0.15	0.14	−0.21	0.20	−0.22
His	0.03	−0.24	0.14	0.13	−0.21	0.17	−0.22
Ile	0.16	−0.27	0.20	0.05	−0.35^*^	0.06	−0.36^*^
Leu	0.10	−0.27	0.18	0.09	−0.29	0.07	−0.30
Lys	0.32	−0.35^*^	0.21	−0.10	−0.54^**^	0.04	−0.54^**^
Met	0.24	−0.35^*^	0.22	−0.02	−0.44^*^	−0.03	−0.45^*^
Phe	−0.06	−0.27	0.06	0.30	−0.08	0.15	−0.10
Thr	0.17	−0.31	0.19	−0.02	−0.38^*^	0.13	−0.38^*^
Trp	0.16	−0.30	0.02	0.10	−0.34^*^	0.30	−0.34^*^
Val	0.12	−0.30	0.18	0.06	−0.33^*^	0.13	−0.34^*^
Nonessential amino acids							
Ala	0.14	−0.33^*^	0.15	0.06	−0.36^*^	0.15	−0.37^*^
Asp	0.14	−0.30	0.16	0.05	−0.36^*^	0.14	−0.37^*^
Cys	0.19	−0.29	0.09	0.09	−0.37^*^	0.09	−0.38^*^
Glu	0.08	−0.25	0.24	0.08	−0.26	0.01	−0.28
Gly	0.03	−0.31	0.11	0.19	−0.22	0.19	−0.24
Pro	0.21	−0.31	0.25	−0.10	−0.40^*^	−0.16	−0.43^*^
Ser	0.13	−0.29	0.20	0.04	−0.34^*^	0.11	−0.35^*^
Tyr	−0.28	−0.19	−0.04	0.48^**^	0.18	0.22	0.16

CP, crude protein; ST, starch; EE, ether extract; CF, crude fiber; NDF, neutral detergent fiber; ADF, acid detergent fiber; Arg, Arginine; His, Histidine; Ile, Isoleucine; Leu, Leucine; Lys, Lysine; Met, Methionine; Phe, Phenylalanine; Thr, Threonine; Trp, Tryptophan; Val, Valine; Ala, Alanine; Asp, Aspartic acid; Cys, Cystine; Glu, Glutamic acid; Gly, Glycine; Pro, Proline; Ser, Serine; Tyr, Tyrosine.

* p<0.05,

** p<0.01.

**Table 9:** Prediction equations of standardized ileal digestibility of amino acid based on the chemical composition of wheat bran samples in 13-day-old broilers (%, as DM basis)

Prediction equations	R^2^	p-value
Essential amino acids		
SID Arg = 119.43–2.77CP–3.88ash	0.86	<0.01
SID His = 151.82–3.29CP–8.57ash+0.22NDF	0.86	<0.01
SID Ile = 233.30–12.40EE–1.50NDF–5.03CP–4.48ash	0.85	<0.01
SID Leu = 14.76+6.70CF–5.18ADF	0.57	<0.01
SID Lys = 11.00+1.81NDF–10.44ash	0.69	<0.01
SID Met = 82.47+16.86CF–12.71ADF+1.75CP–14.88ash–11.28EE	0.89	<0.01
SID Phe = 166.87–4.66CP–1.40NDF–3.48EE+3.19ash	0.94	<0.01
SID Thr = 75.49–2.72CP–5.07EE	0.53	<0.01
SID Trp = 38.47+8.22ash–2.23ADF+0.81NDF–3.13CP	0.78	<0.01
SID Val = 195.47–4.47CP–1.13NDF–8.32EE–3.39ash	0.81	<0.01
Nonessential amino acids		
SID Ala = 71.547–2.205CP–4.264ADF+4.192CF	0.56	<0.01
SID Asp = 170.235–5.163CP–6.212EE–1.143ADF–3.841ash	0.77	<0.01
SID Cys = 4.645+6.745CF+2.451CP–3.733ADF–4.299ash–4.872EE	0.94	<0.01
SID Glu = 242.563+0.394CF–1.362NDF-8.79EE–6.999ash–3.795CP	0.97	<0.01
SID Gly = 144.843–4.233CP–4.513ash–0.625ADF	0.73	<0.01
SID Pro = 290.03–1.974NDF–12.211EE–6.255ash–4.890CP	0.89	<0.01
SID Ser = 253.775–13.818EE–1.535NDF–5.690CP–5.023ash	0.86	<0.01
SID Tyr = –61.144–1.429CP–0.985NDF+19.209ash+10.799EE+1.033ADF	0.92	<0.01

DM, dry matter; SID, standard ileal digestibility; CP, crude protein; NDF, neutral detergent fiber; EE, ether extract; CF, crude fiber; ADF, acid detergent fiber; Arg, Arginine; His, Histidine; Ile, Isoleucine; Leu, Leucine; Lys, Lysine; Met, Methionine; Phe, Phenylalanine; Thr, Threonine; Trp, Tryptophan; Val, Valine; Ala, Alanine; Asp, Aspartic acid; Cys, Cystine; Glu, Glutamic acid; Gly, Glycine; Pro, Proline; Ser, Serine; Tyr, Tyrosine.

**Table 10:** Prediction equations of standardized ileal digestibility of amino acid based on the chemical composition of wheat bran samples in 28-day-old broilers (%, as DM basis)

Prediction equations	R^2^	p-value
Essential amino acids		
SID Ile = 240.24–4.10ADF–7.02CP	0.27	0.01
SID Lys = –208.63–5.84ADF+3.59NDF+27.02EE+20.99ash	0.74	<0.01
SID Met = 231.66–3.97ADF–6.33CP	0.36	<0.01
SID Thr = –2.70–4.53ADF+3.38NDF+25.88EE+18.53ash	0.60	<0.01
Nonessential amino acids		
SID Ala = 220.56–6.52ADF-9.02CP+1.92NDF	0.48	<0.01
SID Asp = –216.14–4.77ADF+3.23NDF+27.12EE+22.95ash	0.71	<0.01
SID Cys = –91.10–3.12ADF+16.00ash+14.47EE+1.60NDF	0.53	<0.01
SID Pro = 158.36–2.04ADF–3.33CP	0.33	<0.01
SID Tyr = –279.66+34.49ash+21.50EE+4.72CP	0.49	<0.01

DM, dry matter; SID, standard ileal digestibility; ADF, acid detergent fiber; CP, crude protein; NDF, neutral detergent fiber; EE, ether extract; Ile, Isoleucine; Lys, Lysine; Met, Methionine; Thr, Threonine; Ala, Alanine; Asp, Aspartic acid; Cys, Cystine; Pro, Proline; Tyr, Tyrosine.
